# Fissure-first bilobectomy of a giant lung abscess combined with a squamous cell carcinoma via a minimally invasive open surgery

**DOI:** 10.1186/s40792-021-01308-2

**Published:** 2021-10-16

**Authors:** Suiha Uchiyama, Shuhei Iizuka, Toru Nakamura

**Affiliations:** Department of General Thoracic Surgery, Seirei Hamamatsu General Hospital, 2-12-12, Sumiyoshi, Naka-ku, Hamamatsu, Shizuoka 430-8558 Japan

**Keywords:** Lung cancer, Fissureless lobectomy, Lung abscess

## Abstract

**Background:**

Fissureless lobectomies are beneficial for preventing prolonged air leaks (PALs). Despite the widespread use of this technique in lobectomy cases, there have been no reports on fissureless bilobectomies to date.

**Case presentation:**

A 73-year-old man with an 80-pack per year smoking history was diagnosed with a stage 1 primary squamous cell carcinoma in the right lower lobe. He developed a lung abscess inside the tumor 6 weeks after the cancer diagnosis and a surgical resection was planned. A middle and lower bilobectomy was mandatory because of the interlobar pulmonary artery involvement. We chose a fissureless technique to avoid any cancer dissemination and bacterial spillage. The thoracoscopic view revealed that the tumor volume was too large to flexibly mobilize. The minimally invasive open surgery (MIOS) approach was valuable in that it combined direct vision and a thoracoscopic maneuver for treating even a large, distended mass. He was discharged uneventfully 9 days after the operation.

**Conclusions:**

The fissureless bilobectomy, in addition to preventing PALs, was a feasible option for preventing cancer dissemination and bacterial spillage for a lung abscess. The MIOS was a safe and minimally invasive approach for even a giant abscess that inhibited the flexible mobilization of the lung.

## Background

A prolonged air leak (PAL) after a pulmonary resection is a significant risk factor of a longer drainage period and length of the hospital stay (LOS) [1]. A fissureless lobectomy is beneficial for preventing air leaks by dividing the fused lung parenchyma with staplers in cases with a fused fissure or emphysema [2, 3].

There are two distinct approaches for the fissureless lobectomy. One is a fissure-last lobectomy with the hilar structures being divided first and the fissures being divided in the last step, and the other is a fissure-first lobectomy with a reversed procedure [4, 5]. Despite the widespread use of this technique in lobectomy cases, to the best of our knowledge, there has been no report on a fissureless bilobectomy to date. We herein report a successful bilobectomy in a case with a giant lung abscess combined with a squamous cell carcinoma and describe the technical significance especially regarding the minimally invasive open surgery (MIOS) [6, 7].

## Case presentation

**Fig. 1:**
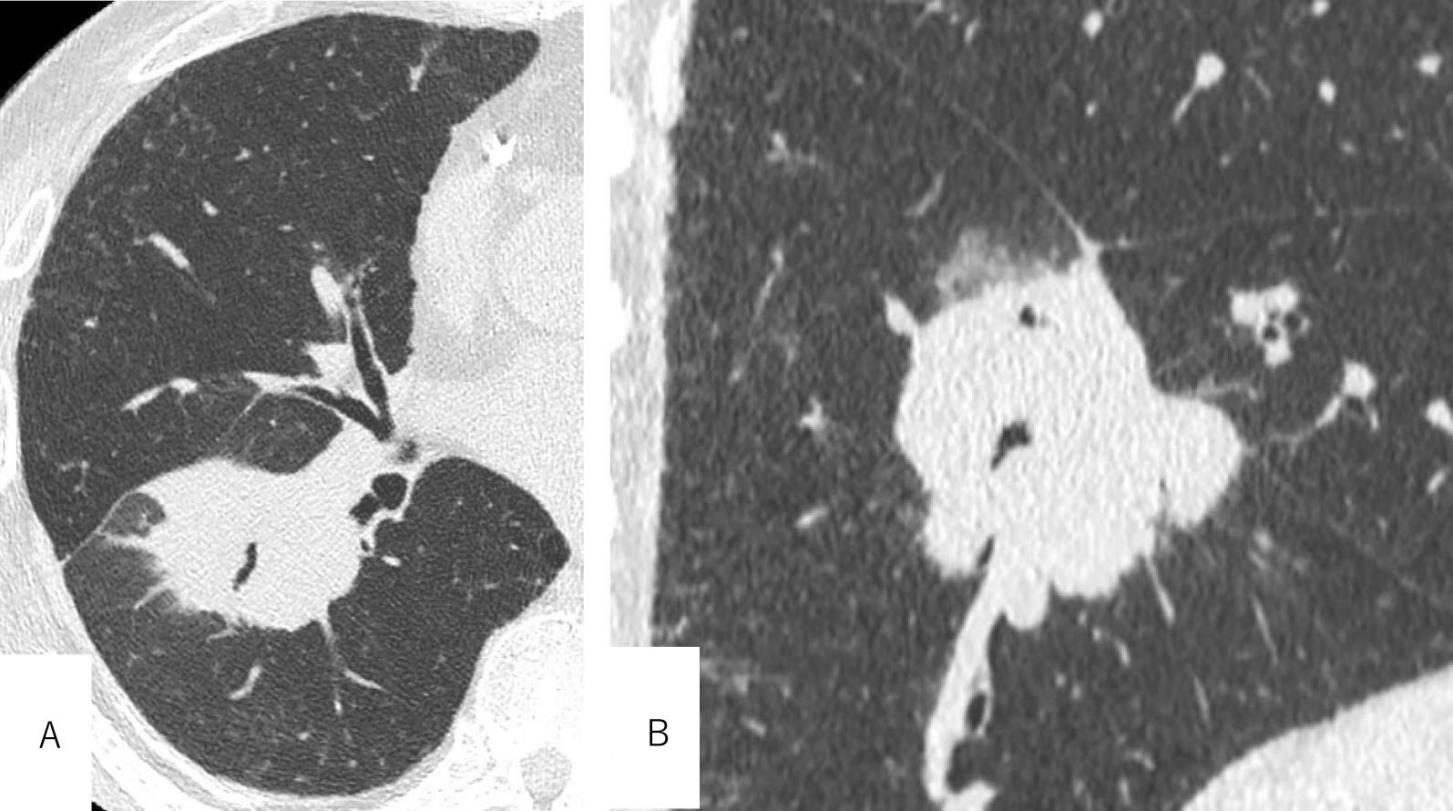
**A** The initial CT showed a lung mass infiltrating the interlobar PA in the right lower lobe. **B** A sagittal view revealed that the lung mass was located in the basal segment

A 73-year-old man with an 80-pack per year smoking history, emphysema, and poorly controlled diabetes presented with a lung mass and was diagnosed with a stage 1 primary squamous cell carcinoma (Fig. 1).

**Fig. 2:**
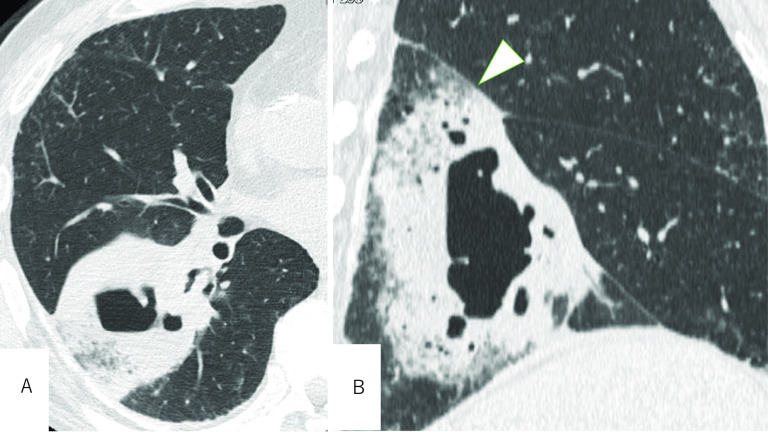
**A** An additional CT 6 weeks later showed a distended cavity formation. **B** A sagittal view revealed the abscess cavity occupying the entire lower lobe adjacent to the oblique fissure (arrowhead)

He developed a febrile illness due to an accompanying lung abscess inside the tumor 6 weeks after the cancer diagnosis. Chest computed tomography (CT) revealed a distended cavity formation occupying the entire lower lobe adjacent to the oblique fissure (Fig. 2). A surgical resection was planned and a middle and lower bilobectomy was mandatory because of the interlobar pulmonary artery (PA) involvement.

The thoracoscopic findings revealed a fibropurulent organized phase empyema that required a thoracoscopic adhesiotomy and decortication. The lower lobe was markedly distended and flexible mobility was lacking even after the total adhesiotomy. A posterolateral thoracotomy with an 8-cm skin incision was made to harvest an intercostal muscle pedicle on the 5th rib. Both the oblique and horizontal fissures were completely fused due to both the inflamed lung abscess and surrounding empyema. We chose the fissureless technique not only to avoid alveolar leakage, but also cancer dissemination and bacterial spillage.

**Fig. 3:**
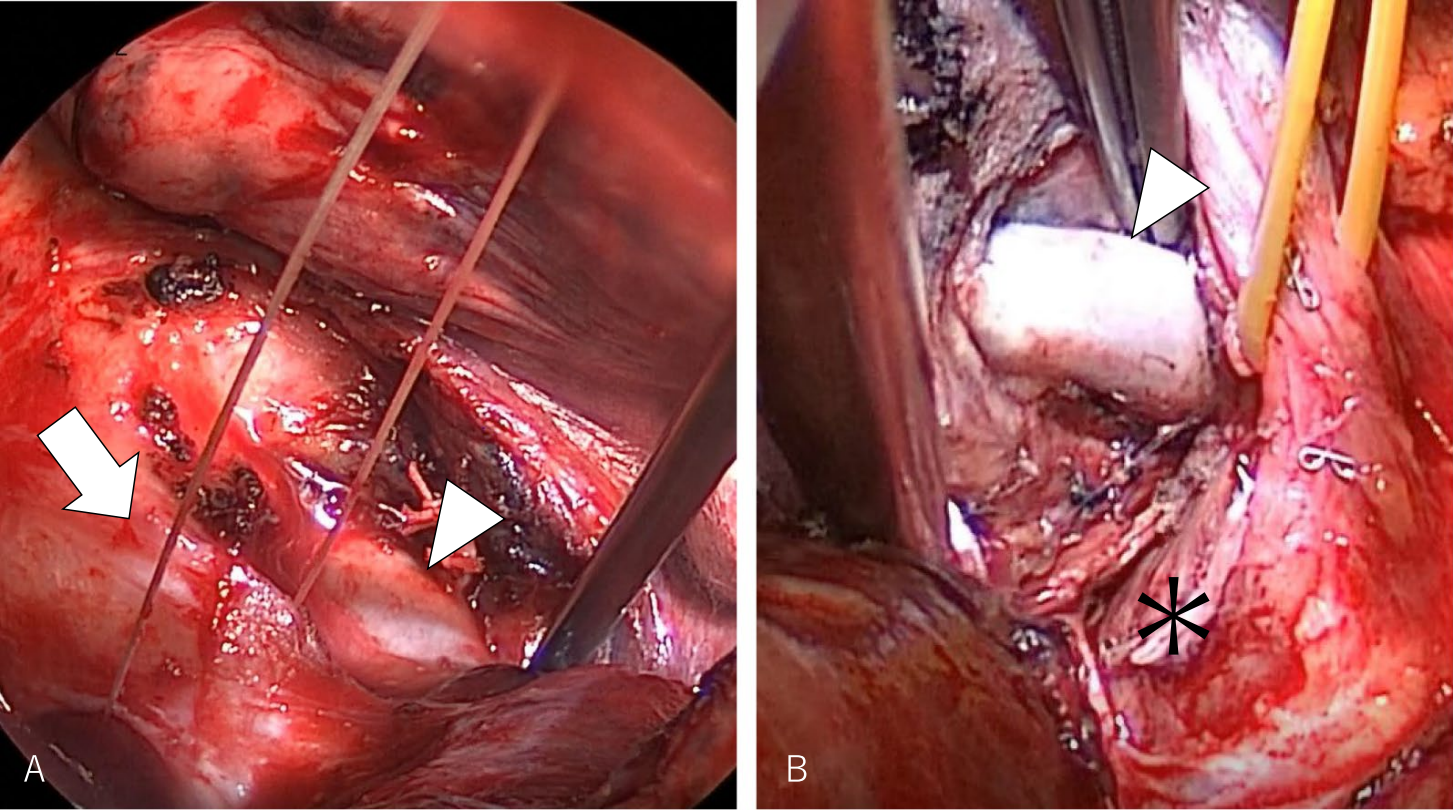
**A** The interlobar lymph nodes were removed posteriorly to expose the posterior aspect of the interlobar PA (arrowhead). The intermediate bronchial trunk was tugged by a silk thread (arrow). **B** The anterior aspect of the interlobar PA (arrowhead) was exposed by dividing the middle lobe vein (asterisk) anteriorly. The upper pulmonary vein was retracted by a silicone thread. Bidirectional exposure of the interlobar PA allowed for a safe tunnel dissection of the fused fissure

The interlobar lymph nodes of the posterior hilum (#11 s) [8] were removed to expose the PA, which was dissected from the intermediate bronchial trunk (Fig. 3A). These procedures allowed performing the fissure-last bilobectomy by initially transecting the bronchus [9]. However, the tumor volume was too large to expose the remnant hilar structures even after transecting the intermediate bronchial trunk, and therefore we altered the plan and performed a fissure-first lobectomy. To divide the fused fissure, the anterior aspect of the PA was exposed by transecting the middle lobe vein anteriorly (Fig. 3B). This bidirectional exposure of the interlobar PA enabled a safe tunnel dissection of the fused fissures and facilitated dividing the fissures with staples.

The exposed interlobar PA, bronchial trunk, and lower lobe vein were transected sequentially to complete the bilobectomy. The resected specimen was contained within a surgical bag and retrieved through the 8-cm incision. The bronchial stump was covered with the harvested intercostal muscle flap. The operative time was 392 min with an estimated blood loss of 830 mL. He was uneventfully discharged 9 days after the operation.

### Discussion

A PAL after a lung resection could prolong the LOS and tube duration and increase infectious complications such as pneumonia or empyema [10, 11]. A fissureless lobectomy is a useful approach to prevent air leakage by dividing the lung parenchyma with a stapler. It has been applied to a wide variety of lung cancer surgeries [4, 5, 12, 13], but a fissureless bilobectomy has never been reported in the literature.

The present case was at a high-risk for a PAL because of the fused fissures with underlying emphysema.

Furthermore, a conventional fissure dissection might have led to the dissemination of the cancer and bacterial spillage because the infected tumor was located close to the fused fissure. We applied the fissureless technique to resolve those problems.

We initially intended to apply the fissure-last technique by first transecting the intermediate bronchus. However, the lower lobe with the distended abscess cavity was too large to flexibly mobilize and expose the remnant hilar structures even after transecting the bronchus. These findings led us to perform a fissure-first lobectomy by first dividing the fused fissures.

The most significant pitfall of the fissureless lobectomy is PA injury [5]. The preceding interlobar lymphadenectomy at the posterior hilum was essential to expose the posterior aspect of the interlobar PA in the present case [9]. Dividing the interlobar PA and bronchial trunk posteriorly also convinced us of both the resectability and feasibility of the fissureless technique at as early a stage of the surgery as possible. A subsequent transection of the middle lobe veins exposed the anterior aspect of the interlobar PA and facilitated a safe bidirectional tunnel dissection of the fused fissures.

We employed the MIOS approach because at least an 8-cm thoracotomy was required to retrieve the resected specimen. Part of the bidirectional manipulation described above was executed thoracoscopically because of the poor direct view due to the large lung mass. Combining the direct view and thoracoscopic maneuver allowed for a high quality and safe surgery with a minimal skin incision and resulted in a satisfactory outcome. The present case suggested that the fissureless approach was applicable for a bilobectomy, and the MIOS, a small open chest surgery with an endoscopic view, was a feasible option even for a large volume inflammatory lung disease.

## Conclusions

A fissureless bilobectomy for a lung abscess combined with cancer is a feasible option for preventing cancer dissemination and bacterial spillage in addition to a PAL. The preceding interlobar lymphadenectomy posteriorly is essential to avoid injury to the PA and to facilitate a safe tunnel dissection of the fused fissures. The MIOS is a safe and minimally invasive approach even for a giant abscess, which inhibits the flexible mobilization of the lung.

## Data Availability

Not applicable.

## Abbreviations

PAL: Prolonged air leak
MIOS: Minimally invasive open surgery
LOS: Length of the hospital stay
CT: Computed tomography
PA: Pulmonary artery
